# Electroconvulsive therapy for depression and oral dyskinesia in a patient who developed parkinsonism induced by valbenazine

**DOI:** 10.1002/pcn5.70135

**Published:** 2025-06-10

**Authors:** Yuhei Mori, Yuhei Suzuki, Akiko Sato, Risa Shishido, Yuri Kobayashi, Yuta Fukuchi, Shota Inada, Yuka Iwasaki, Riko Sato, Keitaro Takada, Naoki Morita, Itaru Miura

**Affiliations:** ^1^ Department of Neuropsychiatry, School of Medicine Fukushima Medical University Fukushima Japan

**Keywords:** depression, dyskinesia, electroconvulsive therapy, parkinsonism, valbenazine

## Abstract

**Background:**

Tardive dyskinesia (TD) is a movement disorder related to long‐term antipsychotic use and characterized by involuntary repetitive movements that often affect the oral and facial muscles. Although valbenazine, a vesicular monoamine transporter 2 (VMAT2) inhibitor, is an effective treatment of TD, it may induce drug‐induced parkinsonism (DIP) in some patients. Electroconvulsive therapy (ECT) improves depressive symptoms and certain movement disorders; however, its role in managing TD, particularly in patients intolerant to VMAT2 inhibitors because of DIP, remains elusive.

**Case Presentation:**

Herein, we describe a 65‐year‐old Japanese woman with a history of major depressive episodes and oral dyskinesia. The patient was treated with valbenazine for oral dyskinesia; however, she developed DIP, requiring valbenazine discontinuation. After the worsening of depression, the patient was hospitalized and underwent 10 sessions of modified ECT, which remarkably helped improving depressive symptoms and oral dyskinesia.

**Conclusion:**

This case suggests that ECT could be a viable treatment option for managing patients with depression and oral dyskinesia who are susceptible of valbenazine‐induced parkinsonism.

## BACKGROUND

Tardive dyskinesia (TD) is a persistent movement disorder linked to long‐term use of dopamine receptor‐blocking agents, specifically: antipsychotics. It is characterized by involuntary, repetitive movements that most commonly affect the oral and facial muscles but may also involve movements of the limbs and trunk. TD pathophysiology is not completely understood but is thought to result from dopamine receptor supersensitivity caused by chronic blockade of dopamine.^1^

The primary pharmacological treatment of TD involves inhibitors of the vesicular monoamine transporter 2 (VMAT2), such as valbenazine and deutetrabenazine, which decrease TD symptoms by modulating dopamine release.^2^ However, these agents can cause drug‐induced parkinsonism (DIP), particularly in older patients or those with pre‐existing parkinsonian features, limiting their use.^3^, ^4^ Among the pharmacological strategies, switching to clozapine, an antipsychotic with lower affinity to dopamine D2 receptors, has been reported to improve symptoms in some cases of TD. However, the supporting evidence is moderate and primarily reached in patients with treatment‐resistant schizophrenia. In contrast, using GABAergic agents and dietary supplements for TD is supported by lower levels of evidence.^2^ Given these limitations, alternative treatments are required for managing patients intolerant to VMAT2 inhibitors.

Electroconvulsive therapy (ECT), known for its efficacy in treatment‐resistant depression, has also demonstrated benefits in certain movement disorders, including parkinsonism and dystonia, possibly through its effects on neuroplasticity and neurotransmitter modulation.^5^, ^6^ Although reports of ECT improving parkinsonism and catatonia exist, its role in patients with TD remains elusive.

Herein, we present the case of a 65‐year‐old Japanese woman with recurrent major depressive episodes and oral dyskinesia. The patient developed DIP following valbenazine treatment of TD, which necessitated discontinuation. Following the worsening of depressive symptoms, the patient was hospitalized and underwent ECT, which considerably improved the depression and TD symptoms. This case suggests that ECT is a viable option for managing patients with TD and depression who cannot tolerate VMAT2 inhibitors. Written informed consent for publication was obtained from the patient and their family. This study followed the Ethical Guidelines of the Fukushima Medical University Hospital and conformed to the Declaration of Helsinki.

## CASE PRESENTATION

A 69‐year‐old Japanese woman presented with major depressive disorder (recurrent episodes, severe psychotic features). The patient reported no history of smoking, alcohol or drug dependence, or physical illness. At 59 years of age, the patient experienced the onset of depressive symptoms without an identifiable trigger, including depressed mood, motivation loss, reduced appetite, and insomnia. The patient was admitted to our hospital when the symptoms worsened. On admission, mirtazapine was administered. However, the patient's condition deteriorated to a state of depressive stupor, rendering oral intake nearly impossible. Consequently, mirtazapine was discontinued and intravenous clomipramine was administered. Despite the treatment, the patient's symptoms remained refractory, therefore we performed ECT. The patient responded well to 10 ECT sessions, resulting in a significant improvement in the depressive symptoms. Subsequently, maintenance therapy with amoxapine (50 mg/day) was administered and the patient's condition remained stable without relapse until approximately 68 years of age. However, around 67 years of age, the patient developed oral dyskinesia, which was suspected to be linked to amoxapine use. Since amoxapine had been discontinued from the market in Japan and the patient had experienced no depressive relapse for almost 10 years, it was withdrawn at around 68 years of age, but oral dyskinesia persisted. The involuntary movements were confined to the orobuccal region and were persistent rather than sudden or transient in nature, and tic disorders were considered unlikely based on clinical observation. Additionally, a dental evaluation revealed no oromandibular abnormalities. At that time, the severity of dyskinesia was rated as 6 on the Abnormal Involuntary Mobement Scale (AIMS). Although the patient's dyskinesia was mild, she expressed a strong desire for symptom improvement.

**Figure 1:**
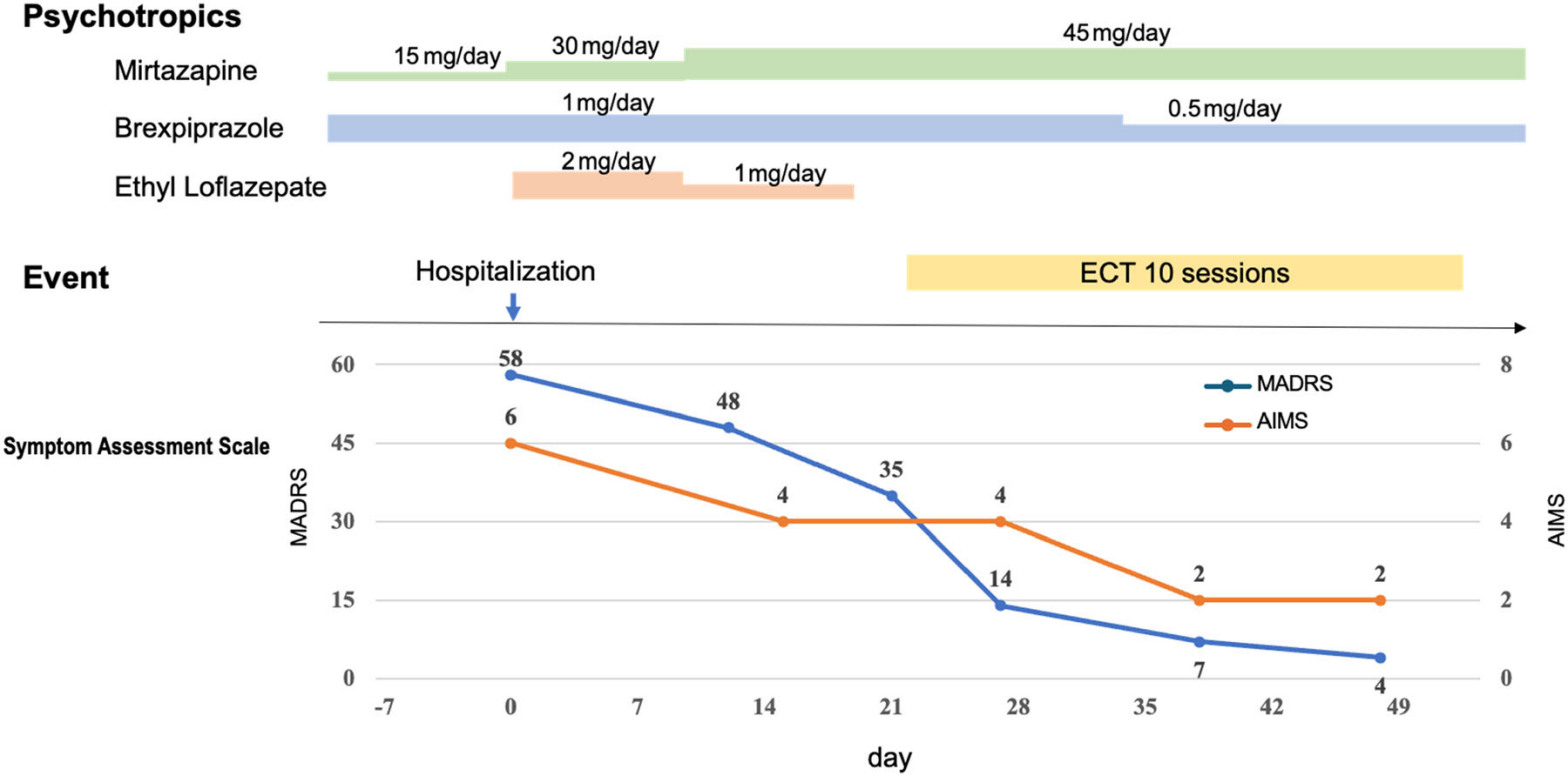
Treatment course and changes in several symptom assessment scales. Hospital day 0 represents the day of admission. Montgomery Åsberg Depression Rating Scale (MADRS) and Abnormal Involuntary Mobement Scale (AIMS) scores decreased following electroconvulsive therapy (ECT). MADRS and AIMS are total scores.

At the age of 69 years, valbenazine treatment was initiated at a dosage of 40 mg/day and titrated to 80 mg/day. However, the patient subsequently developed parkinsonian symptoms, including bradykinesia and hand tremors. The patient was referred to the neurology department, wherein DIP was suspected, resulting in valbenazine discontinuation. The patient's Parkinsonian symptoms resolved approximately 5 days following cessation. Dopamine transporter single‐photon emission computed tomography (DaTSCAN) was performed to assist in the differential diagnosis, which revealed no evidence of striatal dopamine transporter depletion. DaTSCAN was performed to assist in the differential diagnosis, which revealed no evidence of striatal dopamine transporter depletion. In addition, I‐metaiodobenzylguanidine (MIBG) myocardial scintigraphy was performed and showed no significant abnormalities. DIP was diagnosed based on the emergence of extrapyramidal symptoms shortly after valbenazine initiation, absence of Parkinsonian features prior to treatment, and complete symptom resolution following drug discontinuation. In addition, DaTSCAN and MIBG myocardial scintigraphy revealed no abnormalities, further supporting the diagnosis of DIP over a neurodegenerative condition. Three months later, the patient experienced a recurrence of depressive symptoms, including depressed mood, anxiety, agitation, fatigue, appetite loss, and insomnia. Furthermore, the patient developed hypochondriacal delusions, believing that her tongue had turned white and deformed. Given the presence of psychotic symptoms, the patient was diagnosed with major depressive disorder (recurrent episodes and severe psychotic features) according to the Diagnostic and Statistical Manual of Mental Disorders, Fifth Edition. The patient was subsequently readmitted to our hospital. Blood sampling and magnetic resonance imaging of the patient's head showed no other abnormalities. The patient had already been on mirtazapine (15 mg/day) for approximately 1 week as an outpatient. The dose was gradually increased to 15 mg/week until it reached 45 mg/day. Additionally, ethyl loflazepate and brexpiprazole were administered due to severe anxiety and agitation. Mirtazapine was selected considering the patient's prominent anxiety and agitation, as well as its low affinity to dopamine receptors. Although the potential risk of exacerbating TD was considered with brexpiprazole, it was chosen due to the presence of psychotic symptoms and its partial agonist activity at dopamine D2 receptors, which might offer a protective effect against TD compared with other second‐generation antipsychotics. Although we considered waiting for the effects of the medication, ECT was initiated 3 weeks following admission for the following reasons: the patient's previous positive response to ECT and the presence of valbenazine‐refractory oral dyskinesia. Ten ECT sessions were conducted; the patient's symptoms were assessed using the Montgomery–Åsberg Depression Rating Scale (MADRS). Bitemporal electrodes were then placed. The Thymatron device settings were as follows: 0.9‐A current, 0.5‐ms pulse width, 30‐Hz stimulation frequency, and 6.5‐s stimulus duration, which were standard at our parameters. Ten sessions were performed, determined based on symptom improvement and standard clinical protocol. The initial stimulus dose was set at 35% using the half‐age method and increased to 40% from the fifth session onward. In cases of insufficient seizure under the half‐age method, it is generally recommended to increase the stimulus dose to approximately 1.5 times the initial setting (e.g., 50%–55%). However, considering the risk of cognitive side effects in this elderly patient, we adopted a more conservative approach and increased the dose only to 40% from the fifth session onward. Adequate generalized seizures were confirmed in all sessions except for the fourth. After three ECT sessions, the patient's depressive symptoms improved. Depressed mood, motivation loss, reduced appetite, and insomnia gradually alleviated. The patient's oral dyskinesia began to improve after around three sessions. The MADRS score had improved to 4 (Figure 1). Furthermore, AIMS improved to 2. No significant adverse events were noted during the ECT. Thereafter, the patient was maintained on mirtazapine therapy and remained stable during outpatient follow‐up.

## DISCUSSION

To the best of our knowledge, this is the first report to assess ECT's effectiveness in treatment‐resistant oral dyskinesia and depression in a patient with a history of valbenazine‐induced parkinsonism. The patient had been administered amoxapine for approximately 10 years. Amoxapine is a tricyclic antidepressant with dopamine receptor‐blocking properties.^7^ Additionally, long‐term dopamine receptor blockade was presumed to have resulted in oral dyskinesia development. In this case, the emergence of DIP after valbenazine use suggests dopaminergic system vulnerability. Although DaTSCAN and MIBG myocardial scintigraphy showed no abnormalities, ongoing monitoring for potential neurodegenerative progression remains important. VMAT2 inhibitors, such as valbenazine, might induce parkinsonism by disrupting dopamine storage and increasing oxidative stress.^8^, ^9^, ^10^ These findings highlight the protective role of VMAT2 and potential risks associated with its inhibition. ECT might have improved symptoms by stabilizing the patient's dysregulated dopaminergic system. Prior studies suggest that ECT modulates dopamine release and receptor sensitivity in the striatum, promoting dopaminergic homeostasis rather than simply enhancing transmission.^11^ Additionally, ECT has been shown to increase dopamine transporter and VMAT2 binding in animal models, potentially reducing dopaminergic stress and improving motor symptoms.^12^ Although previous studies have suggested that switching to clozapine may benefit some cases of TD due to its lower dopamine D2 receptor affinity, in the present case clozapine was not considered an appropriate option due to the absence of psychotic symptoms and diagnosis of depression rather than schizophrenia. Previous case reports and reviews have suggested that ECT may be beneficial for TD in patients with comorbid treatment‐resistant depression, long‐term antipsychotic use, or limited pharmacologic options. The present case shared these clinical characteristics, making ECT a reasonable therapeutic option under the constraints posed by valbenazine‐induced parkinsonism.^6^ Overall, this case underscores ECT's potentials as both an antidepressant and neuromodulatory treatment in patients with dopaminergic vulnerability, particularly when pharmacological options are limited.

Although ECT was the primary intervention, the potential influence of concomitant medications warrants consideration. Mirtazapine, through antagonism of 5‐HT2C and α2‐adrenergic receptors, may alter dopaminergic tone, contributing to dyskinesia in susceptible individuals.^13^ However, its high affinity to 5‐HT2A receptors has also been associated with antidyskinetic effects in preclinical studies.^14^ Ethyl loflazepate likely played only a minor sedative role. Brexpiprazole, a D2 partial agonist, is generally associated with dopaminergic stabilization but has been linked to rare cases of TD.^15^, ^16^, ^17^ Thus, although ECT was likely responsible for the observed improvement, adjunctive pharmacotherapy might have had modulatory effects.

However, our study has some limitations. Given that a single case has been described, the results cannot be generalized to broader populations, and further studies are necessary. The exact biological mechanism by which ECT alleviates oral dyskinesia remains elusive because no direct evaluation of dopamine receptor function has been conducted. Although drug‐induced dyskinesia was strongly suspected based on the medication history and clinical course, we acknowledge that a definitive differentiation from spontaneous dyskinesia in an older patient might be difficult. Another limitation is that the therapeutic effects of ECT might not be permanent; the possibility of relapse or recurrence cannot be excluded. Furthermore, considering that the patient exhibited DIP following valbenazine treatment, the case might represent a vulnerable phenotype, and future recurrence patterns may differ from those typically observed in conventional cases of TD. Anxiety symptoms related to depression might also have exacerbated the dyskinesia. Furthermore, the clinical findings supported the diagnosis of DIP; however, the potential underlying neurodegenerative processes cannot be entirely excluded. Finally, as discussed above, the roles of mirtazapine and brexpiprazole in symptom improvement have been examined, but their contribution cannot be fully ruled out and should still be considered a limitation.

## CONCLUSION

This case demonstrated ECT's efficacy in depression and valbenazine–refractory oral dyskinesia. ECT resulted in a substantial improvement in depressive symptoms and complete oral dyskinesia resolution. ECT may be considered a viable therapeutic option for managing refractory oral dyskinesia, particularly when pharmacological treatment is limited.

## AUTHOR CONTRIBUTIONS

Yuhei Mori designed the study, contributed to the interpretation of results, and drafted the manuscript and figure. Yuhei Mori, Yuhei Suzuki, Akiko Sato, Risa Shishido, Yuri Kobayashi, Yuta Fukuchi, Shota Inada, Yuka Iwasaki, Riko Sato, Keitaro Takada, and Naoki Morita treated the patients. Itaru Miura supervised the work. All authors have reviewed the manuscript and approved its submission.

## CONFLICT OF INTEREST STATEMENT

The authors declare no conflict of interest.

## ETHICS APPROVAL STATEMENT

The Committee of Fukushima Medical University approved this study.

## PATIENT CONSENT STATEMENT

The patient provided written informed consent for the publication of this report.

## CLINICAL TRIAL REGISTRATION

N/A.

## Data Availability

Data sharing not applicable to this article as no datasets were generated or analysed during the current study.
